# Meath Hospital, Dublin

**Published:** 1831-04-01

**Authors:** 


### MEATH HOSPITAL, DUBLIN.

On painful Swellings of the Lower Extremities. By Drs. Graves and Stokes.

Pursuing our account of the 5th vol. of the Dublin Hospital Reports, we come to the above subject, which is interesting at the present time, when the pathology of phlegmatia dolens is so much discussed.

*Case.* Eliza O'Donnel, aged 21, was admitted on the 3d of June, 1829, with symptoms of gastric fever, and severe pain in the right side under the ribs. Thirty leeches afforded some relief, with aperient medicine. 6th. Convalescent —menstruation scanty. 7th. Had been kept awake all night by pain in the left leg, which was swollen, tender, hot, tense, but not red nor pitting on pressure. The tenderness was greatest along the course of the saphena vein, which felt like a cord. Pulse was 108 and hard—tongue brown and dry—much thirst. Twenty leeches were applied along the course of the saphena vein—calomel and opium—hip-bath. The next day she was greatly relieved; and, in a few days, her mouth was affected by the mercury, and the pain subsided in the limb, which had decreased considerably in size, was less tense, *and pitted on pressure.*

" In this case a decided tendency to a recurrence of the affection was observed, and the pain, tenderness, and swelling returned several times, but in a less severe form, and generally yielded to the application of leeches and stupes. The pain in one attack was greatest in the calf of the leg, in another at the instep, and in a third at the middle of the thigh. The disease in the left leg, after continuing with intermissions for three weeks, subsided, leaving, however, the limb considerably swollen, and the patient in a state of emaciation and exhaustion.

On the 28th of June, a nearly similar train of local symptoms commenced in the right leg, attended with so much constitutional irritation and debility, that we were apprehensive of the result.

The violence of the symptoms, however, subsided after the application of twenty leeches, and the use of remedies hereafter to be mentioned, so that, although her convalescence was tedious, the local symptoms had disappeared before the middle of July."

It is worthy of remark that, when leeches were applied during the swollen state of the limb, a large quantity of serous fluid flowed from the bites, before any red blood made its appearance. During this state also, the swelling of the member was by no means uniform, often simulating most perfectly the fluctuation caused by an abscess immediately under the integuments. The following remarks are particularly interesting at the present time.

"An accurate observation of numerous cases both of phlegmasia dolens occurring after delivery, and of painful swelling of the extremities appearing during or after fever,* has satisfied us of the pathological identity of the two diseases. In both œdema occurs, unattended by redness, but accompanied by increase of heat, with great tenderness and pain, and followed for a considerable time by impaired motion of the limb.

In both diseases the swelling and other symptoms are frequently not confined to any one portion of the extremity, but extend uniformly over the leg and thigh. In both diseases, however, we have also often observed, that the pain, heat, and swelling, occupied particular parts of the limb, while the rest was comparatively free from disease. Thus in some cases a portion of the thigh was intensely engaged, while the leg and foot remained free, and after some days the diseased action seemed to change its place, and successively attacked the other portions of the limb, without, however, any precise order in the mode of succession. In consequence of this, our treatment has been directed to different portions of the limb, according to the situation of the disease; and we constantly found that the degree of swelling in the part attacked was proportioned to the accompanying heat, pain, and tenderness. In some cases we have observed this affection to be attended by a cordy and painful state of the saphena vein, proving that it participated in the disease; but as this state of the vein, where it did occur, was in some cases subsequent to the disease of the other parts of the limb; and as in the majority of our cases of phlegmasia dolens, and in the painful swelling of the extremity after fever in the male and female subject, no such affection of the saphena occurred, we think that the latter cannot in justice be considered as the cause of the disease. The occasional occurrence of the swelling in the inferior portion of the limb in the first instance, and its erratic nature, militate against the idea that the disease proceeds from an affection of the large venous trunks; and in two instances we have seen the disease desert its original seat, and concentrate itself in the knee-joint, producing obstinate inflammation of the part, which in one case, that of a male after fever, terminated in anchylosis, and in the other, that of a female who laboured under true phlegmasia dolens after delivery, the same unfortunate result was with difficulty arrested.

As the latter occurrence, and many obvious considerations, leave little doubt concerning the inflammatory nature of

* Dr. Tweedie, Edinburgh Medical and Surgical Journal. No. 97.

the disease; it remains to be considered what are the parts engaged. To us it would appear that *the subcutaneous cellular tissue is primarily affected,* sometimes generally, at others partially. It is not unusual to meet with cases either of general or local anasarca evidently of an inflammatory origin, accompanied by pain and heat, but unattended by redness. The external and vascular layer of the corium remaining uninflamed, will account for the absence of redness in this disease, as well as in the inflammatory anasarca.

The cellular tissue seems to follow the same law as serous membranes. Moderately inflamed it effuses an unusual quantity of its natural secretion, *serum.* When the irritation is more intense, the effusion is also altered; it contains more animal matter, approaching in its qualities to coagulable lymph, and sometimes it is of a puriform nature. It is to be remarked, that suppuration has occasionally been observed in phlegmasia dolens when very intense, but usually the effusion appears to be the result of an intermediate degree of inflammation between that which produces puriform and merely serous effusion. The swelling is consequently more apt to produce by means of the coagulable matter contained in the effusion, considerable hardness arising from the consolidation of the cellular tissue. A state of parts not observed in the beginning or termination of the disease, when the inflammation is more moderate, which we have frequently pointed out to our pupils both in phlegmasia dolens and the painful swelling of the extremity after fever. Did space permit, further arguments might be adduced from considering the pathology of Barbadoes leg, and the disease termed berri berri in Ceylon."

In respect to treatment, our authors depend chiefly on fomentations, leeches, narcotics—and after the inflammatory symptoms have subsided, they use bandages, tonics, and diuretics. When the knee is attacked, frequent leechings, blisterings, and the inclined plane are necessary. Salivation is questionable.

A case occurred subsequent to the above, which we shall put on record, *without abridgement,* because it militates against the doctrine which we have always maintained respecting phlegmasia dolens. We never withhold facts that seem to run counter to our own theories.

"A young man of a strong habit was employed for two successive days in working in a ditch, and was consequently obliged to stand in water above his knees during that time. On the following day he became affected with lassitude, vertigo, and general weakness, and complained of severe pain in the right thigh. These symptoms continued for seven days, when he was admitted into the Meath Hospital.

On admission his countenance was anxious and depressed; the tongue furred; thirst; headach; urine scanty, turbid, and high-coloured; pulse 96; skin mottled with petechiæ. In addition to these general symptoms his respiration was observed to be laboured and unequal, with some cough; face very livid. But his chief complaint was a severe pain in the upper and anterior portion of the right thigh, which was greatly aggravated by motion or pressure. He had also severe pain in the left hypochondrium, increased by inspiration or cough.

At this time no swelling whatever of the limb could be detected; but in the course of two days the upper portion of the thigh became evidently swollen, the part being extremely tender, elastic, but not at all red. The pain of the side continued, and extensive bronchial and pneumonic inflammation was detected. General bleeding, and very free leeching to the limb, was employed. The blood was not inflammatory, and no relief was experienced by the patient. The swelling of the thigh increased; calomel and opium were freely exhibited, but without any effect. The typhoid symptoms increased, and the patient died on the fourth day after his admission.

On dissection we found the right lower extremity swollen, and tense in its superior portion, while the leg and foot were slightly anasarcous. The sac of the pericardium contained some sero-purulent fluid, and that portion covering the auricles and great vessels was vascular, and in many places covered with coagulable lymph. Both lungs were in a state of extreme sanguineous congestion, with commencing solidity in their postero-inferior portion, and general inflammation of the pleura, as shewn by a reticular exudation of coagulable lymph, which occurred in greatest quantity in the most inferior portions. The bronchial mucous membrane was universally red, and the tubes filled with frothy mucus. No disease could be detected in the lining membrane of the heart. The right ventricle contained a fibrinous coagulum of the usual appearance. The gastro-intestinal system appeared remarkably healthy, except in the great extremity of the stomach, where the mucous membrane presented a dotted red colour. The spleen enlarged, flabby and pale, was found in a state of purulent infiltration, bearing some resemblance to the lung in the third stage of acute pneumonia.

The vena cava contained a few portions of a substance of a granular appearance, friable and of a yellowish colour. These did not adhere to the vessel, which otherwise appeared healthy. In the external iliac vein, however, we found, just above Poupart's ligament, a large concretion of a similar nature, nearly plugging up the vessel, and extending into some of the minute collateral branches. The lining membrane was red, and in one point adhered to the coagulum. No puriform matter could be detected. The femoral and popliteal veins were healthy, as also the arteries. The cellular tissue of the limb was pale and œdematous.

It cannot be denied that this case is strongly corroborative of the opinion before entertained, and lately insisted on by Tommasini, that the phlegmasia alba dolens is in reality owing to phlebitis."

Setting aside the petechial fever, and the numerous inflammatory affections found in various parts of the body, the fact of the disease proving fatal in eleven days is so totally at variance with general experience in phlegmasia dolens, that this single fact alone is quite sufficient to destroy all supposed identity of the two diseases. We appeal to every practitioner of any experience as to the justice of this remark.

We return the talented reporters many thanks for these valuable cases.

### II. GANGRENOUS ERYSIPELAS.

James Keogh, a sawyer, æt. 34, became a patient of Mr. M'Namara in the Meath Hospital, in consequence of gangrenous erysipelas, which destroyed the whole of the integuments of the arm, forearm, and a portion of those of the hand; he was suffering from hectic in an extreme degree, with a quick, small, and very irregular pulse; indeed so far were the powers of life reduced, that the proposal of removing the arm at the shoulder-joint appeared to many a very hazardous measure, an opinion that, I confess, I agreed in, seeing how small a quantity of blood the loss of which would be sufficient to deprive such a patient of life, an opinion that was strengthened by the length and severity of two operations I had seen elsewhere performed.

The patient was carried into the operating theatre in his bed, on Saturday, the 18th of September last, and having been placed sitting on the side of it, Mr. M'Namara commenced the operation, by making a flap of the deltoid muscle, in the manner recommended by De La Faye; he then cut into the joint, dislocated the bone, and finished the operation by bringing out his knife behind the bone, making the inferior flap by one stroke of the instrument. The axillary artery, the circumflex arteries, and one or two inconsiderable vessels, were now secured, and the flaps were brought together by means of adhesive plaster and sutures. An anodyne draught was administered, though I could not say that the patient suffered extreme pain either during the operation or after it; indeed his sufferings were of so short duration, the cutting part occupying but a minute and a half, that I think I am warranted in saying, that amputation at the shoulder-joint is attended with as little pain as a common amputation, and (if I am to judge from the present case, which undoubtedly was a good specimen of Irish surgery), with certainly less hæmorrhage, in so much that more than two ounces of blood were not lost on the occasion. It is also worthy of remark, that the axillary vein did not bleed.

It is unnecessary to occupy your valuable space by a detailed account of the treatment, which did not embrace any thing out of the ordinary course pursued in such cases; the man has hourly gained strength; the ligatures have all come away; and the wound is now, ten days from the operation, nearly healed. There are one or two observations with which I shall trouble you on the present case; and first, it is obvious how much the debility here may, relatively, be produced by the presence of an extensively suppurating surface, over which the constitution was incapable of exerting any salutary control; secondly, how little we should permit ourselves to be influenced by this apparent debility, which will certainly disappear as soon as its cause is removed; thirdly, how unnecessary the tying of the axillary artery or vein is, as a preliminary step to the performance of the operation; and, lastly, how uncalled for is the removal of the cartilage from the glenoid cavity, a practice which has been adopted from the fear of the occurrence of subsequent inflammation and disease, which, from a reference to this and other cases, I am warranted in saying never occur.

### III. Edœma of the Larynx—Tracheotomy.

William Kenny, aged 47, of a bilious temperament, admitted on the 16th Sept. stated, that about three o'clock on the evening before, he had been attacked by a rigor, which was succeeded by sore throat; that previous to this he had always enjoyed good health, with the exception of a slight pulmonary catarrh of some years' standing; that at midnight he awoke, unable to swallow; his breathing extremely difficult, noisy, and accompanied by a slight cough. For the relief of these symptoms he used a muriatic acid gargle. Present state: voice husky in the highest degree; breathing deep, slow, remarkably loud, and attended with considerable muco-sibilous râle, cough, accompanied with mucous expectoration; deglutition impossible, as far as regards solids, and a considerable portion of any fluid was even regurgitated through the nares; the larynx very tender upon pressure; 28 ounces of blood were immediately drawn from the arm, and he was ordered to take two grains of tartarized antimony every hour in solution. Noon; breathing less loud; pulse 128, soft; skin moist; has vomited frequently some bilious matter; his condition, however, is not improved. Seven o'clock, p.m.; oppression of breathing so great, that the patient expressed himself incapable of existing; cough troublesome; stridulous respiration increased considerably; pulse quick; extremities cold; upper part of the body bedewed with sweat; integuments of the neck considerably swollen from œdema; deglutition entirely obstructed. On examining the fauces, Mr. M'Namara discovered a tumour of the size of a large walnut, formed by the integuments of the epiglottis, which had become œdematous, and from its situation capable of completely obstructing deglutition whenever the patient made an effort to swallow; it did not, however, obstruct respiration, inasmuch as it stood erect in the fauces, and could not be laid down upon the rima glottidis; it however followed the motion of the tongue, and thus became an impediment to deglutition when that organ was moved backwards, occupying the pharynx, rendering it impossible for food to pass downwards. Considering that the integuments of the other parts of the larynx were in the same state, and the man must be suffocated if some effort were not made to admit air to the lungs, Mr. M'Namara determined upon performing the operation of tracheotomy immediately. The patient having been properly placed, and the usual incision made in the integuments, the trachea was now laid bare at a depth of fully two inches and a half below the surface; to such an extent had the œdema proceeded; the tube was easily perforated, but on endeavouring to turn the knife in order to enlarge the wound by dividing the cartilages, he found it impossible, without risking the breaking of the instrument, as they were ossified; he therefore enlarged the wound with a pair of scissors, the rings of the trachea giving audible evidence of their ossification on being cut through; there was not any bleeding of consequence during the operation, and the man was put to bed expressing how completely he felt himself relieved. He was ordered four grains of calomel every second hour, and to inhale the steam of warm water during the night.

17th. The patient breathed partly through the wound and by the larynx; the croupy sound of respiration lessened, and deglutition gradually improved; swelling of the epiglottis much less; pulse 112; respiration 24; skin moist; bowels moved; no thirst; tongue clean, and no appearance of ptyalism, though half a drachm of calomel had been taken during the night. Repeat the calomel. A silver tube was placed in the wound.

18th. The respiration was natural; the tongue clean; pulse 90; bowels moved three times; the epiglottis has subsided to its natural size; integuments of the neck also much less; had some sleep during the night; the calomel was now discontinued, and he was ordered a draught, consisting of an ounce of the infusion of roses, a drachm of Epsom salts, and the same of tincture of jalap.

It is unnecessary so report further on this case, inasmuch as he is rapidly improving, complaining of no inconvenience but what results from the soreness of his mouth. The tube he occasionally introduces himself, and there is every reason to think that he will be able shortly to relinquish it altogether; nor need I impress upon you, Mr. Editor, the advantage of an early operation in cases of œdema of the larynx.*

The second case exemplifies the good effects of tracheotomy in saving life, when any sudden inflammatory affection obstructs the air-passage. We have seen several lives sacrificed from the fear of performing this operation, which, though not without its difficulties, is perfectly free from danger.

---

* Reported by Mr. Beetham, in No. 377 of the Lancet.

